# Inhibition of non-canonical NF-κB signaling suppresses periodontal inflammation and bone loss

**DOI:** 10.3389/fimmu.2023.1179007

**Published:** 2023-04-18

**Authors:** Tsukasa Aoki, Fumitaka Hiura, Aonan Li, Nan Yang, Nana Takakura-Hino, Satoru Mukai, Miho Matsuda, Fusanori Nishimura, Eijiro Jimi

**Affiliations:** ^1^ Laboratory of Molecular and Cellular Biochemistry, Division of Oral Biological Sciences, Faculty of Dental Science, Kyushu University, Fukuoka, Japan; ^2^ Department of Periodontology, Division of Oral Rehabilitation, Faculty of Dental Science, Kyushu University, Fukuoka, Japan; ^3^ Department of Health and Nutrition Care, Faculty of Allied Health Sciences, University of East Asia, Shimonoseki, Japan; ^4^ Oral Health/Brain Health/Total Health Research Center, Faculty of Dental Science, Kyushu University, Fukuoka, Japan

**Keywords:** nuclear factor-k B, periodonititis, osteoclasts (OCs), inflammation, bone, NIK

## Abstract

Periodontal disease is an infectious disease that affects many people worldwide. Disease progression destroys the alveolar bone and causes tooth loss. We have previously shown that alymphoplasia (*aly/aly*) mice harboring a loss-of-function mutation in the *map3k14* gene, which is involved in p100 to p52 processing of the alternative NF-κB pathway, exhibited mild osteopetrosis due to decreased number of osteoclasts, suggesting the alternative NF-κB pathway as a potential drug target for the amelioration of bone disease. In the present study, wild-type (WT) and *aly/aly* mice were subjected to silk ligation to establish a periodontitis model. Alveolar bone resorption was suppressed in *aly/aly* mice by decreased numbers of osteoclasts in the alveolar bone in comparison to WT mice. Furthermore, the expression of receptor activator of NF-κB ligand (RANKL) and TNFα (cytokines involved in osteoclast induction in periligative gingival tissue) was decreased. When primary osteoblasts (POBs) and bone marrow cells (BMCs) derived from WT and *aly/aly* mice were prepared and co-cultured, osteoclasts were induced from WT-derived BMCs, regardless of the origin of the POBs, but hardly formed from *aly/aly* mouse-derived BMCs. Furthermore, the local administration of an NIK inhibitor, Cpd33, inhibited osteoclast formation and thereby inhibited alveolar bone resorption in the periodontitis model. Therefore, the NIK-mediated NF-κB alternative pathway can be a therapeutic target for periodontal disease.

## Introduction

Periodontal disease is an inflammatory disease caused by periodontal pathogens (1–3). As inflammation progresses, the alveolar bone is destroyed and the disease becomes chronic. Periodontal tissue possesses anatomical features and immune mechanisms that protect against invasion by periodontal pathogens (1–3). When bacterial plaque adheres to the gingival sulcus for a long time, lymphocytes infiltrate and inflammation occurs in the gingiva. The invasion of periodontal tissue by periodontal pathogens leads to infiltration by lymphocytes, neutrophils, macrophages, and plasma cells, the disappearance of collagen fibers, and the subsequent expansion of inflammation. Cellular components of periodontal pathogens, proteases, bacterial toxins, and proteases, cytokines, and inflammatory chemical mediators released by host cells, expand inflammatory infiltration in the apical and lateral directions, leading to the formation of periodontal pockets and the destruction of alveolar bone (1–3). Excessive alveolar bone resorption leads to tooth loss and significantly impacts quality of life (QOL) (1–3).

Osteoclasts are essential for alveolar bone resorption in periodontitis (4). Osteoclast precursor cells derived from hematopoietic stem cells differentiate into osteoclasts (4, 5). In periodontitis, lipopolysaccharide (LPS) produced by periodontal pathogens, and inflammatory cytokines produced by host immune cells act on the periodontal ligament cells and osteoblasts to induce the receptor activator of nuclear factor κB (NF-κB) ligand (RANKL) to differentiate into osteoclasts (4, 6). When RANKL binds to RANK expressed on the plasma membrane of osteoclast progenitors, NF-κB and mitogen-activated protein kinase (MAPK) are activated within the cells, and NFATc1, a master regulator of osteoclastogenesis, resulting in osteoclast differentiation (7). In addition, since NF-κB regulates the induction of inflammatory cytokine genes, NF-κB are therapeutic targets for diseases associated with inflammatory bone destruction, including rheumatoid arthritis (7).

There are two pathways for NF-κB activation. One is a so-called “classical pathway” that is activated shortly after stimulation with inflammatory cytokines (e.g., tumor necrosis factor-α [TNF-α] and interleukin-1β [IL-1β]) accompanied by IκBα degradation (7, 8). The other is a so-called “alternative pathway” that is activated several hours after stimulation with cytokines involved in the development of lymph nodes (e.g., CD40L and lymphotoxin-β [LTβ]) mediated by the NF-κB inducing kinase (NIK)-IκB kinase α (IKKα) pathway (7–9). RANKL activates these two pathways, and mice with a double knockout of the two important subunits of each pathway (NF-κB1 and NF-κB2) exhibit osteopetrosis a total lack of osteoclasts (7, 10, 11). These indicate that both NF-κB activation pathways play an important role in RNAKL-induced osteoclast bone resorption.

We previously showed that lymphoid hypoplasia (*aly/aly*) mice with a natural loss-of-function mutation in the *map3k14* gene, which encodes a kinase essential for p100 to p52 processing in the NF-κB alternative pathway, exhibit osteopetrosis in association with decreased osteoclast numbers (12, 13). Furthermore, stimulation of osteoclast precursors from *aly/aly* mice with RANKL suppressed the expression of NFATc1 due to the inhibition of p100 to p52 processing. Compound 33 (Cpd33), which was developed as a selective inhibitor of NIK, inhibited RANKL-induced osteoclastogenesis in a dose-dependent manner without affecting cell viability (14). Moreover, Cdp33 treatment inhibited osteoclast formation, thereby suppressing bone loss in ovariectomized mice (14). These results suggest that the alternative NF-κB pathway is a suitable therapeutic target for diseases with excessive bone loss. In this study, we used *aly/aly* mice and Cpd33 to investigate the possibility that the alternative pathway of NF-κB could be a therapeutic target for periodontal disease.

## Materials and methods

### Reagents

Glutathione S-transferase (GST)-RANKL and recombinant human macrophage-stimulating factor (M-CSF) were purchased from Oriental Yeast Company, Ltd. (Shiga, Japan) and FUJIFILM Wako Pure Chemical Corporation (Osaka, Japan), respectively. LPS (Escherichia coli 055: B5) was purchased from Sigma-Aldrich (St Louis, MO, USA). Cpd33 was kindly provided by Genentech Inc. (South San Francisco, CA, USA).

### Mice

Mouse handling and all procedures were approved by the Animal Care Committee of Kyushu University, according to the guidelines of the Japanese Council on Animal Care (Approval Number: A21-284-3, A22-233-2). All experiments were performed in accordance with ARRIVE guidelines. Eight-week-old male C57BL/6J and *aly/aly* mice were purchased from CLEA Japan (Tokyo, Japan).

### Mouse periodontal disease model

To induce periodontal bone loss in 8-week-old C57BL6/J (wild-type:WT) and *aly/aly* mice, 6-0 silk ligatures (Akiyama Medical Mfg. Co., Ltd., Tokyo, Japan) were tied around the right maxillary second molar (15). Meanwhile, the contralateral molar of each mouse was left non-ligated (baseline control for bone loss measurements). In some experiments, C57BL6/J mice were divided into two groups, a control group (treated with phosphate-buffered saline [PBS]) and a Cpd33-treated group (0.5 mg/kg dissolved in DMSO, diluted with PBS). Ligation followed by a Hamilton syringe with a D33 gauge needle (Hamilton Company, Reno, NV, USA) was used to inject 10 μl of DMSO with PBS or Cpd33 into the palatal gingiva of the ligated second maxillary molar 3 times for 7 days. The ligation remained intact in all mice for the duration of the experiment. The mice were euthanized after 7 days and the maxilla was removed for a further analysis.

### Radiological assesments

**Figure 1:**
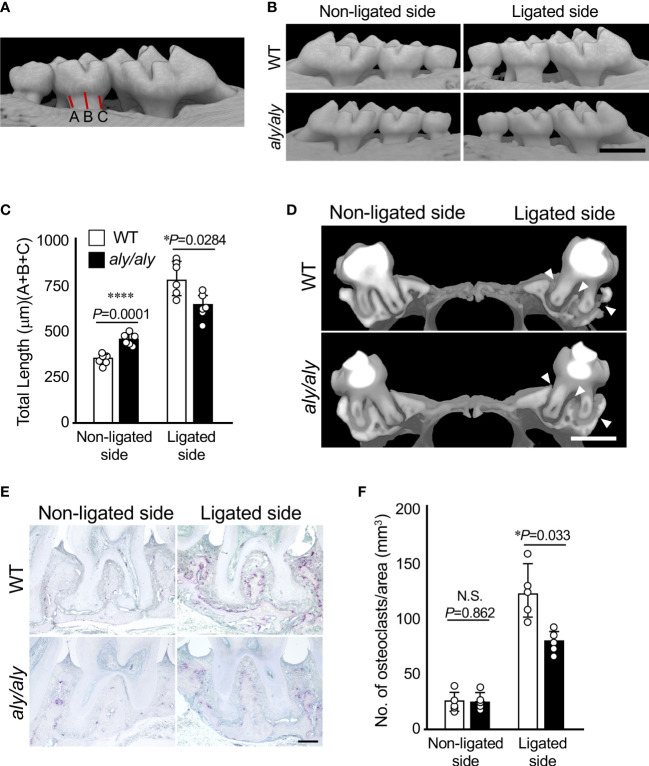
Alveolar bone resorption was inhibited in a ligation-induced periodontitis model in *aly/aly* mice. **(A)** Measured distance from CEJ to alveolar bone was shown. **(B)** 6-0 silk ligature was ligated around the right maxillary second molar of C57BL/6 (WT) and *aly/aly* mice for 7 days. **(B)** Representative three-dimensional μCT images of the maxilla of WT or *aly/aly* mice 7 days after ligation. Scale bar = 1  mm. **(C)** The distance from the cement-enamel junction (CEJ) to the apex of alveolar bone (AB) on µCT images was measured using an image analysis software program. Data are expressed as the mean ± SD (n = 6). **(D)** Representative three-dimensional μCT images of the mid-to-sagittal section of a maxillary second molar of WT or *aly/aly* mice. Scale bar = 1  mm. **(E)** Bone resorption was confirmed by the detection of osteoclasts by TRAP staining. Scale bar = 200 μm. **(F)** The number of TRAP-positive osteoclasts per area (mm^2^) was counted. TRAP-positive MNCs were counted from her two random coronal sections of ligated molars of five male mice in each group. Data are expressed as the mean ± SD (n = 5). *p < 0.05, ****p < 0.0001. N.S., not significant.

Excised maxillae were fixed in 4% paraformaldehyde in PBS (pH 7.4) at 4°C for 24 h, and then washed several times with ice-cold PBS. Bone mineral density (BMD) and three-dimensional (3D) reconstruction images of the maxilla were acquired using microfocal computed tomography (μCT; ScanXmate-L090T, Comscan Co, Kanagawa, Japan). Alveolar bone resorption was calculated by measuring the distance from the cement/enamel junction (CEJ) to the alveolar bone surface at three locations, mesial, central, and distal, of the maxillary second molars (**Figure 1A**), and expressed as the mean value.

### Histrogical analysis

After the completion of μCT imaging, maxillae were decalcified with Osteosoft (Merck, Darmstadt, Germany), sagittal paraffin sections (thickness: 5 μm) were prepared, and then tartrate-resistant acid phosphatase (TRAP) staining was performed to detect osteoclasts. Nuclei were stained with methyl green. Each tissue section was observed using a BZ-9000 (Keyence), and TRAP^+^ multinucleated cells (MNCs) containing ≥ 3 nuclei on the bone surface were counted as osteoclasts.

### Quantitative real-time polymerase -chain reaction

The palatal gingiva of the mouse maxillary second molar on the ligated side and the non-ligated side was cut into 1-mm squares, and total RNA was extracted using ISOGEN II (Nippon Gene, Tokyo) according to the protocol. Total RNA from cultured osteoblasts was extracted using TRIZOL (Invitrogen, Waltham, MA, USA). Total RNA (1 µg) was transcribed into cDNA using a High Capacity cDNA Reverse Transcription Kit (Applied Biosystems, Foster City, CA, USA). Real-time PCR was performed using the KOD SYBR qPCR Mix (Toyobo, Osaka, Japan) using a Thermal Cycler Dice Real Time System II (TaKaRa, Shiga, Japan). The GAPDH was used as an internal control. Primer sequences were as follows: *tnfs11*, 5′- CAGCATCGCTCTGTTCCTGTA -3′ (forward) and 5′- CTGCGTTTTCATGGAGTCTCA -3′ (reverse), *Il-1b*, 5′- TCGCTCAGGGTCACAAGAAA -3′ (forward) and 5′- CATCAGAGGCAAGGAGGAAAAC -3′ (reverse); *Il-6*, 5′- GCTACCAAACTGGATATAATCAGGA -3′ (forward) and 5′- CCAGGTAGCTATGGTACTCCAGAA -3′ (reverse); *tnfa*5′- TCTTCTCATTCCTGCTTGTGG -3′ (forward) and 5′- GGTCTGGGCCATAGAACTGA -3′ (reverse), and *gapdh*, 5′-AACTTTGGCATTGTGGAAGG-3′ (forward) and 5′-ACACATTGGGGGTAGGAACA-3′ (reverse).

### Osteoclastogenesis

Primary osteoblasts (POB) were obtained by the calvaria of 1-day-old neonatal WT or *aly/aly* mice by digesting with 0.1% collagenase (Fujifilm Wako Pure Chemical Industries, Ltd.) and 0.2% dispase (Godo Shusei, Tokyo, Japan). Bone marrow cells (BMCs) obtained from 8-week-old WT or *aly/aly* mice were co-cultured in α-MEM containing 10% fetal bovine serum (FBS), 100 units/ml penicillin, and 100 μg/ml streptomycin, together with or without LPS (100 ng/ml) for 7 days. In some experiments, bone marrow cells obtained from 8-week-old WT or *aly/aly* mice were cultured with M-CSF (50 ng/ml) and RANKL (50 ng/ml) for 5 days. The medium was changed every 2 days. After culturing, the cells were fixed with 3.7% formaldehyde in PBS for 10 min. After permeabilization with ethanol/acetone, cells were stained with TRAP, and TRAP^+^ MNCs were observed under a microscope and counted as osteoclasts.

### Osteoblast differentiation

After the POBs became confluent, they were pretreated with or without Cpd33 (1 μM) and then cultured in osteoblast differentiation medium (α-MEM containing 5% FBS, 10 mM β-glycerophosphate (β-GP), and 50 μg/ml ascorbic acid) in the presence or absence of LPS (100 ng/ml). After fixing the cells with ethanol/acetone on day 7 of culture, they were incubated with alkaline phosphatase (ALP) substrate buffer (10 mg/ml *p*-nitrophenyl phosphate, 0.1 M diethanol amine, and 1 mM MgCl_2_, pH 8.0). After 15 minutes, the reaction was stopped by adding 5M NaOH and then the absorbance at a wavelength of 405 nm was measured with a plate reader (Bio-Rad Laboratories, Inc, Hercules, CA).

### Statistical analysis

Data are presented as the mean ± standard deviation (SD). Means were compared between two groups using the equal variance t-test (versus control; * P < 0.05, ** P < 0.01). Statistical comparisons were performed by an analysis of variance (ANOVA) with Tukey’s test for multiple comparisons, when evaluating more than two groups. *P<0.05, **P<0.01, ***P<0.001, ****P<0.0001. Statistical analysis was performed using the SPSS software package version 27.0 (IBM, Armonk, NY, USA).

## Results

### Alveolar bone resorption in a ligation-induced periodontitis model was inhibited in *aly/aly* mice

First, we established a periodontitis model by ligating the right maxillary second molars of either WT or *aly/aly* mice with silk thread, and examined the degree of alveolar bone resorption at 7 days after ligation using μCT. The distance from the CEJ of the second molar to the alveolar bone surface was shorter in *aly/aly* mice than in WT mice, and the buccal alveolar bone thinning observed in WT mice was suppressed in *aly/aly* mice (**Figures 1B, C**). Alveolar bone resorption on the non-ligated side was significantly longer in *aly/aly* mice than in WT mice (**Figure 1C**). The sagittal analysis of the maxillary second molar region revealed significant thinning of the buccal alveolar bone and significant resorption of the furcation and palatal alveolar bone in the WT mice, whereas these changes were not observed in *aly/aly* mice (**Figure 1D**, arrowheads). Consistent with these results, the number of osteoclasts in the alveolar bone of *aly/aly* mice was lower than that of WT mice in a histological analysis (**Figures 1E, F**). These results indicate that the inhibition of NIK suppresses ligation-induced periodontitis.

### Expression of ligation-induced inflammatory cytokines was suppressed in the gingival tissue of *aly/aly* mice

**Figure 2:**
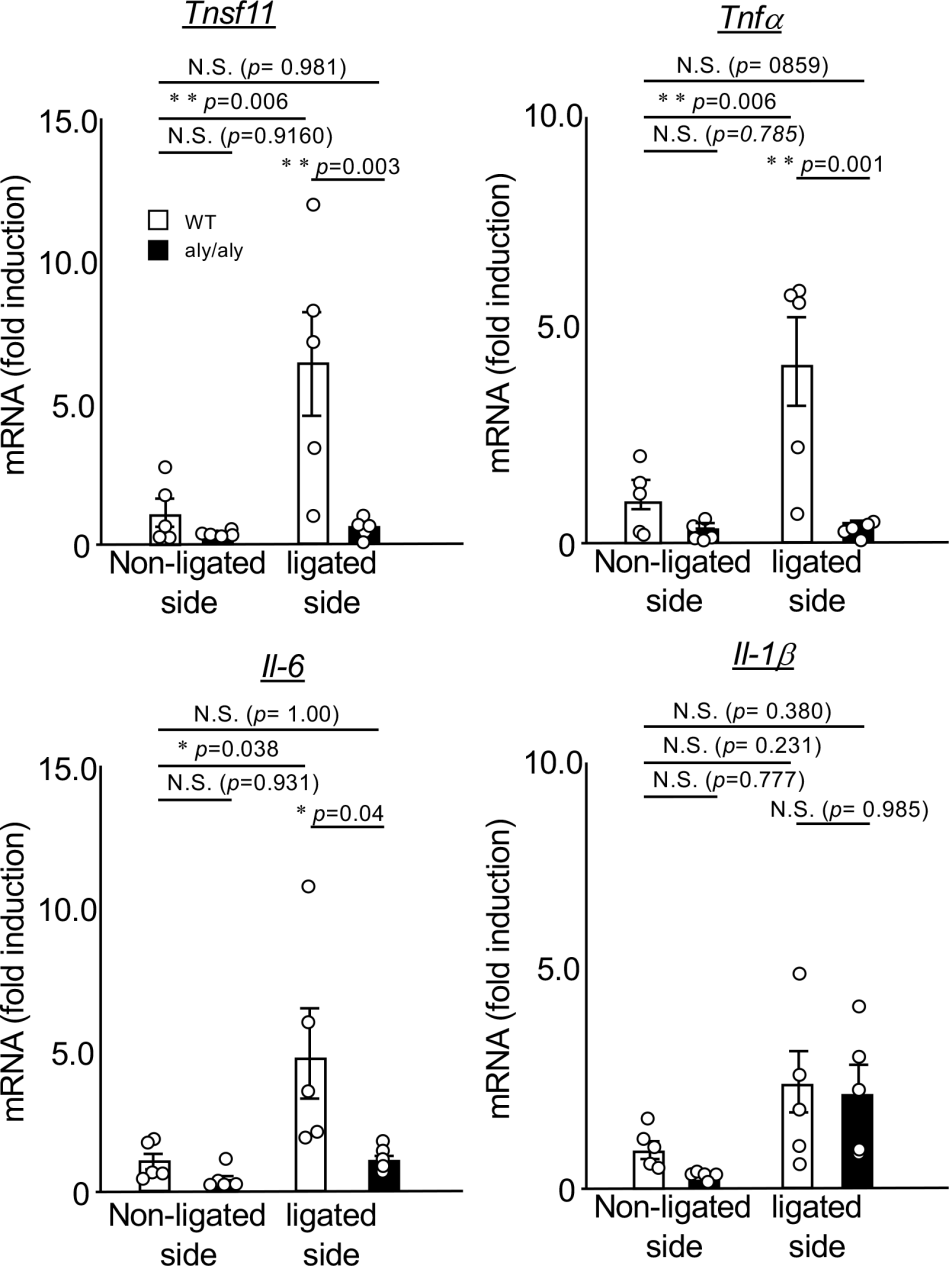
The expression of ligation-induced inflammatory cytokines was suppressed in the gingival tissue of *aly/aly* mice. On the 7th day after ligating the upper right maxillary second molar of WT and *aly/aly* mice, the palatal gingiva of the ligated tooth from each mouse was collected and total RNA was prepared. The mRNA expression of *tnfsf11*, *tnfα*, *IL-6, IL-1β* and *gapdh* was analyzed by real-time polymerase chain reaction. Data are expressed as the mean ± SD (WT:n = 5, *aly/aly*:n = 5). Similar results were obtained from three independent experiments. *P<0.05, **P<0.01. N.S., not significant.

Next, we compared the expression changes of NF-κB-dependent inflammatory cytokines in the palatal gingiva between WT and *aly/al*y mice. In the gingival tissue of *aly/aly* mice, the expression of RANKL, which is essential for osteoclast differentiation, TNFα, and IL-6, which induces osteoclastogenesis (16, 17), were suppressed (**Figure 2**). The expression of IL-1β did not change between the non-ligated side and the ligated side of WT and *aly/aly* mice (**Figure 2**).

### Suppression of osteoclastogenesis in the *aly/aly* mouse periodontitis model was primarily attributed to osteoclast precursors

**Figure 3:**
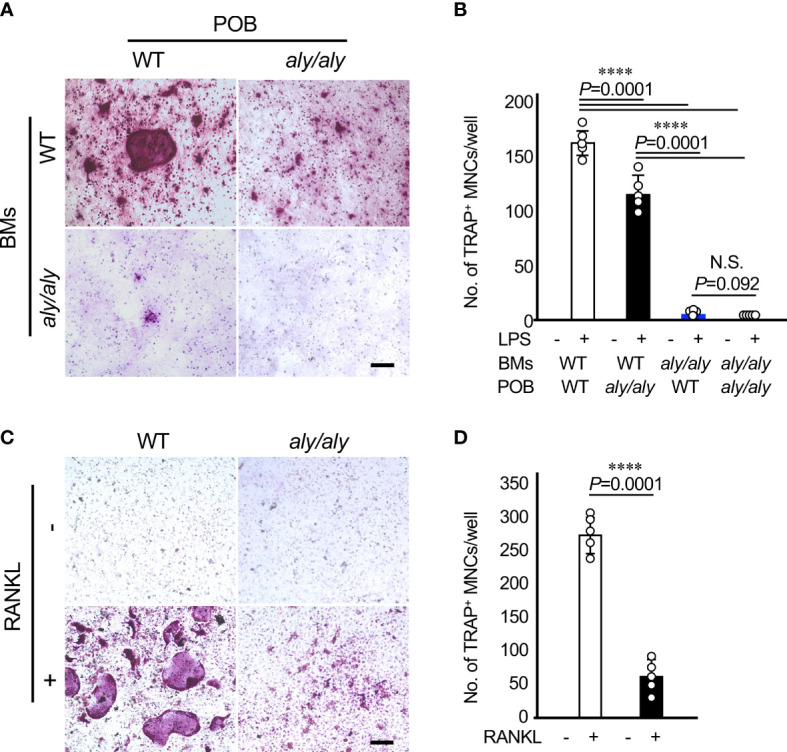
The suppression of osteoclastogenesis in *aly/aly* mice in the periodontitis model was primarily attributed to osteoclast precursors. **(A)** Bone marrow cells (BMCs) and primary osteoblasts (POB) from either WT or *aly/aly* mice were cocultured to differentiate into osteoclasts in the presence or absence of LPS (100 ng/ml) for 7 days. Cells were stained by TRAP. Scale bar = 200 μm. **(B)** The number of TRAP^+^MNCs was counted. Data are expressed as the mean ± SD (n = 5). **(C)** BMCs from either WT or *aly/aly* mice were cultured in the presence or absence of RANKL (50 ng/ml) together with M-CSF (50 ng/ml) for 5 days. Cells were stained by TRAP. Scale bar = 200 μm. **(D)** The number of TRAP^+^MNCs was counted. Data are expressed as the mean ± SD (n = 5). ****P<0.0001. Similar results were obtained from three independent experiments. N.S., not significant.

To clarify the cellular mechanism by which the number of osteoclasts in *aly/aly* mice was reduced in comparison to WT mice in a periodontitis model, primary osteoblasts (POBs) and bone marrow cells (BMCs) from WT and *aly/aly* mice were prepared and cocultured in the presence or absence of LPS. When POBs and BMCs from WT mice and *aly/aly* mice were examined, *aly/aly* mice were found to produce smaller osteoclasts. On the other hand, osteoclasts were hardly detected in the co-culture of BMCs from *aly/aly* mice with POBs from either WT or *aly/aly* mice (**Figures 3A, B**). As we previously reported, the stimulation of BMCs from either WT or *aly/aly* mice with M-CSF and RANKL resulted in the formation of numerous large osteoclasts, whereas a few small osteoclasts were formed in BMCs from *aly/aly* mice (**Figures 3C, D**). These results suggest that the low osteoclast differentiation potential of osteoclast precursors in *aly/aly* mice is mainly responsible for the decrease in the number of osteoclasts in the periodontitis model in comparison to WT mice.

### Cpd33 inhibited the LPS-induced expression of RANKL and inflammatory cytokines in osteoblasts, and restored the LPS-induced suppression of osteoblastic differentiation

**Figure 4:**
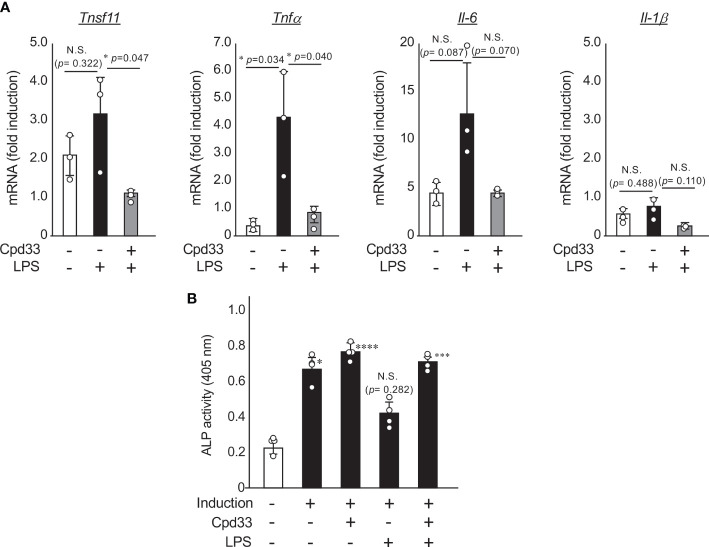
Cpd33 inhibited the LPS-induced expression of RANKL and inflammatory cytokines in osteoblasts, and restored the LPS-induced suppression of osteoblastic differentiation. **(A)** POBs were pretreated with or without Cpd33 (1 μM) for 1h and then treated with LPS (100 ng/ml) for 48 (h) Total RNA was prepared and then subjected to real-time polymerase chain reaction to examine the mRNA expression of *tnfsf11*, *tnfα*, *IL-6, IL-1β* and *gapdh.* Fold-induction was displayed with the expression level of each cytokine before stimulation (time=0) set to 1. Data are expressed as the mean ± SD (n = 3). *P<0.05. **(B)** POBs were cultured with osteoblast inducetion media (αMEM containing 5% FBS, 50 μg/ml ascorbic acid and 10 mM β-grycelophosphate) for 7 days. Pretreatment with or without Cpd33(1 μM) was followed by stimulation with or without LPS (100 ng/ml). After 7 days, alkaline phosphatase (ALP) activity was measured to assess osteoblast differentiation. Absorbance was measured at a wavelength of 405 nm. Data are expressed as the mean ± SD (n = 4). Similar results were obtained from three independent experiments. *P<0.05, ***P<0.001, ****P<0.0001 (vs vehicle). N.S., not significant.

We next investigated the possibility of Cpd33, a selective inhibitor of NIK, as a therapeutic agent for periodontitis. Since we have already reported that Cpd33 suppresses RANKL-induced osteoclastogenesis in a dose-dependent manner (14), we examined the effects of Cpd33 on the induction of RANKL and inflammatory cytokines induced by LPS stimulation in osteoblasts. Cpd33 at a concentration of 1 μM (which completely suppresses RANKL-induced osteoclastogenesis) (14) suppressed the expression of RANKL and TNFα induced by LPS in osteoblasts (**Figure 4A**). Cpd33 tended to suppress LPS-stimulated IL-6 expression, but the difference was not significant. IL-1β levels were almost unchanged after 48 hours of LPS stimulation with or without CPd33 pretreatment. We next investigated the possibility that Cpd33 could restore the suppression of LPS-stimulated osteoblastic differentiation. When POBs were cultured in a medium containing ascorbic acid and β-glycerophosphate and pretreated with Cpd33 for 1 hour before LPS stimulation, the inhibition of ALP activity was restored in comparison to that without pretreatment with Cpd33 (**Figure 4B**). Cpd33 alone slightly increased ALP activity, but the difference was not statistically significant.

### Cpd33 prevented alveolar bone loss in the periodontitis model

**Figure 5:**
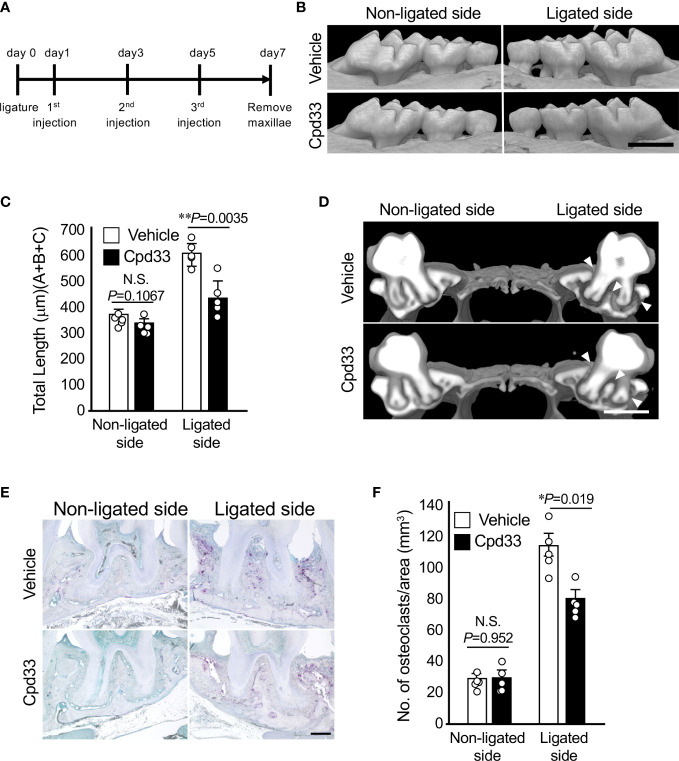
Cpd33 prevented alveolar bone loss in the periodontitis model. **(A)** Experimental schedule was shown. **(B)** 6-0 silk ligature was ligated around the right maxillary second molar of C57BL/6 mice, and from the same day, control group (DMSO) and Cpd33 (0.5 mg/kg) were administered once every two days for 7 days. **(B)** Representative three-dimensional μCT images of the maxilla in each treatment group at 7 days after ligation. Scale bar = 1  mm. **(C)** The distance from the cement-enamel junction (CEJ) to the apex of alveolar bone (AB) on µCT images was measured using an image analysis software program. Data are expressed as the mean ± SD (n = 5). **(D)** Representative three-dimensional μCT images of the mid-to-sagittal section of a maxillary second molar in each treatment group. Scale bar = 1  mm. **(E)** Bone resorption was confirmed by the detection of osteoclasts by TRAP staining. Scale bar = 200 μm. **(F)** The number of TRAP-positive osteoclasts per area (mm^2^) was counted. TRAP-positive MNCs were counted from two random coronal sections of ligated molars of five male mice in each group. Data are expressed as the mean ± SD (n = 10). *p < 0.05, **p < 0.01. N.S., not significant.

We finally examined the possibility of suppressing alveolar bone resorption in a periodontitis model. Cpd33 (0.5 mg/kg) was injected through the palatal gingiva three times a week, and the degree of alveolar bone resorption was evaluated 7 days later (**Figure 5A**). Cpd33 significantly inhibited alveolar bone resorption (**Figures 5B, C**). There was no difference in the amount of alveolar bone resorption on the non-ligated side between the vehicle group and Cpd33-treated group. The sagittal analysis of the maxillary second molar region revealed significant thinning of the buccal alveolar bone and significant resorption of the furcation and palatal alveolar bone in the vehicle group. On the other hand, even in the Cpd33-treated group, thinning of the buccal alveolar bone was observed, while sufficient alveolar bone remained on the furcation and palatal side. (**Figure 5D**). In the histological analysis, the number of osteoclasts in the alveolar bone of Cpd33-treated group was lower than that of the vehicle group (**Figures 5E, F**). These results further supported that the inhibition of NIK suppresses alveolar bone resorption in ligation-induced periodontitis model.

## Discussion

Based on our previous findings that NIK inhibition is effective for suppressing osteoclast differentiation using *aly/aly* mice (12, 13) and the NIK inhibitor Cpd33 (14), the present study demonstrated that NIK inhibition is also effective in alveolar bone resorption, even in an experimental periodontitis model, by inhibiting osteoclastogenesis at histological and cellular levels. When the right upper maxillary second molar of WT mice were ligated using silk thread, osteoclasts appeared and alveolar bone resorption was observed, but the number of osteoclasts was low, and the expression of RANKL and inflammatory cytokines in the gingiva around the ligation was decreased in *aly/aly* mice. The decreases in the number of osteoclasts in the *aly/aly* mouse periodontitis model, were mainly due to cell autonomous defects of osteoclast precursors. Furthermore, the local administration of Cpd33 inhibited alveolar bone resorption by reducing the number of osteoclasts and the expression of inflammatory cytokines, including RANKL, in the surrounding gingiva. in the surrounding gingiva, as seen in *aly/aly* mice. Taken together, NIK can be a suitable target molecule for the treatment of periodontal disease.

As described above, the expression of RANKL in the periligative gingival tissue was suppressed in the *aly/aly* mouse periodontitis model. Thus, we further investigated the possibility that the decrease in the number of osteoclasts observed in the *aly/aly* mouse periodontitis model was caused by the decreased expression of RANKL. POBs and BMCs derived from WT and *aly/aly* mice were prepared, co-cultured in four combinations, and osteoclasts were induced by LPS stimulation. WT mouse-derived BMCs induced osteoclasts in both mouse-derived POBs. However, POBs from aly/aly mice induced smaller osteoclasts than POBs from WT mice. As RANKL expression was decreased in the periligative gingival tissues of *aly/aly* mice, LPS-induced RANKL expression in POB from *aly/aly* mice may suppress compared with POB from WT mice. On the other hand, when BMCs derived from *aly/aly* mice were used, few osteoclasts were formed regardless of whether the POBs were derived from WT or *aly/aly* mice. Furthermore, even when osteoclasts were induced with M-CSF and RANKL using BMCs derived from WT or *aly/aly* mice, only a few osteoclasts were formed from BMCs derived from *aly/aly* mice. These results suggested that RANKL signaling is inhibited in osteoclast progenitor cells derived from *aly/aly* mice. We have already shown that NIK inhibition by *aly/aly* mice and Cpd33 suppresses the RANKL-induced expression of NFATc1 (12–14).

NIK was originally identified as a protein that binds to TRAF2 using the yeast two hybrid system with TRAF2 as a bait (18). Furthermore, the overexpression of NIK activated the canonical pathway of NF-κB (18). On the other hand, Xiao et al. reported that the stimulation of cytokines involved in lymphadenopathy, such as CD40L and BAFF, plays an important role for NIK in the activation of alternative pathways through p100 to p52 processing (19). Despite there seems to be a seemingly clear line between the canonical and alternative NF-κB pathways, NIK may be involved in both. LPS activates canonical NF-κB signaling through TLR4 (20, 21). Among several inflammatory cytokines induced by LPS stimulation, RANKL, TNFαN and IL-6 were decreased in aly/aly mouse-derived or Cpd33-pretreated osteoblasts, whereas, expression of IL-1b was not changed. Since, changes in the expression of inflammatory cytokines, including RANKL, were examined 7 days after ligation and 48 hours after LPS stimulation, detailed time-course changes may yield different results. RANKL is a rare cytokine that activates both the classical and alternative pathways (7), and stimulation of BMCs from aly/aly mice or Cpd33-pretreated BMCs with RANKL did not affect the degradation of IκBα, only p52 processing was inhibited (12–14). Moreover, overexpression of p52 or RelB in BMCs from aly/aly mice induced expression of Cot (cancer ocular thyroid) and NFATc1, thereby rescuing osteoclastogenesis (22). Analysis of the Cot promoter showed that p65 and RelB bind to distal NF-κB binding sites, and that RelB, but not p65, binds to her NF-κB binding sites proximal to her Cot promoter. The alternative pathway is thought to induce osteoclast differentiation through long-term maintenance of Cot expression (22). Thus, the inhibition of alveolar bone resorption in the periodontal disease model by the inhibition of NIK is considered to be mainly due to the inhibition of osteoclast differentiation rather than suppression of inflammation.

From the results of the bone morphometric analysis of the long bones of *aly/aly* mice, the increase in bone mass in *aly/aly* mice was due to not only to the suppression of bone resorption by a decreasing number of osteoclasts, but also to the enhancement of bone formation (12, 13). Furthermore, POBs prepared from *aly/aly* mouse calvaria showed enhanced osteoblastic differentiation in comparison to POBs from WT mice (23). However, the injection of Cpp33 into ovariectomized mice, mainly suppressed bone resorption and had almost no effect on bone formation (14). The reason for this may be that ovariectomized mice are in a state of high turnover where bone resorption and formation are accelerated; thus, the effect of enhancing bone formation may not have been so obvious. In this study, adding Cpd33 to POB from WT mice tended to increase the ALP activity in comparison to the control group, but the difference was not significant. On the other hand, the stimulation of POB with LPS suppressed osteoblastic differentiation, whereas pretreatment with Cpd33 partially restored the suppression of osteoblastic differentiation. These results suggest that the suppression of alveolar bone resorption by *aly/aly* mice and Cpp33 treatment may also involve the effect of preventing the suppression of bone formation under inflammation.

Since the crystal structure analysis of NIK was clarified (24, 25), selective inhibitors of NIK have been developed, and in addition to regulating cell proliferation and differentiation, they are effective against liver inflammation/steatosis, rheumatoid arthritis, lupus, osteoporosis, metabolic disorder, and cancer in animal models (26, 27). NIK-SM1 and NIK inhibitor, 4-(3-((7H-pyrrolo [2,3-d] pyrimidin-4-yl) amino)-4-morpholinophenyl)-2-(thiazol-2-yl) -but-3-yn-2-ol, suppressed alveolar bone resorption in the mouse periodontitis model by inhibiting osteoclast formation and the expression of inflammatory cytokines in the gingiva (28, 29). Injection of LPS into the calvaria of *aly/aly* mice also inhibited osteoclast induction, thereby suppressing bone destruction compared to WT mice (30). In this study, we not only examined the inhibition of alveolar bone resorption by NIK inhibition at the genetic level, but also clarified the structure, cell and histological analysis of the internal alveolar bone. The local administration of Cpd33 to a periodontitis model also inhibited alveolar bone resorption by suppressing osteoclast formation, as in the case of applying a periodontal disease model to *aly/aly* mice. Since we administered Cpd33 locally from the palatal gingiva, Cpd33 could not sufficiently suppress buccal alveolar bone resorption, but Cpd33 was able to suppress palatal and furcation alveolar bone resorption. Since the defect of alveolar bone at the furcation (class II defect) is particularly difficult to regenerate (31), the suppression of alveolar bone resorption at the furcation is considered clinically useful.

As aly/aly mice (32) and NIK-/- mice (33) exhibit abnormal spleen and thymus architecture, complete lack of lymph nodes, and poor antibody production, the long-term administration of inhibitors of NIK might be risk of weakened immune responses. We previously reported that intraperitoneal administration of Cpd33 for 4 weeks in OVX mice inhibited osteoclast formation, thereby suppressing bone loss (14). No significant body weight loss was observed during treatment, and neither structural abnormalities in the spleen and thymus, nor damage to the liver and kidneys were bserved in histological analysis (14). In this study, the local administration of Cpd33, no abnormalities such as gingival redness or necrosis were observed. Furthermore, a small volume of liquid was required for gingival injection, and at concentrations above 0.5 mg/kg, Cpd33 was not sufficiently soluble. In the future, in order to consider the clinical application of Cpd33, it is necessary to investigate in detail the solvent that dissolves Cpd33, the administration method, and the administration period. Taken together, the inhibition of NIK is relatively effective in suppressing alveolar bone resorption due to periodontal disease.

## Data availability statement

The raw data supporting the conclusions of this article will be made available by the authors, without undue reservation.

## Ethics statement

The animal study was reviewed and approved by The Animal Care Committee of Kyushu University.

## Author contributions

The authors TA, and FH performed the experiments, µCT, and histological analysis and prepared the initial version of the paper. AL, NY, and NT-H performed the experiments. EJ designed the study, performed the experiments and the literature review, prepared the initial and the final versions of the paper, and submitted the document. All authors contributed to the article and approved the submitted version.
